# Preoperative Vitamin D Deficiency Is Associated With Higher Vasoactive-Inotropic Scores Following Pediatric Cardiac Surgery in Chinese Children

**DOI:** 10.3389/fped.2021.671289

**Published:** 2021-07-28

**Authors:** Xiuxia Ye, Shumei Dong, Yujiao Deng, Chuan Jiang, Yanting Kong, Lili Tang, Yanlin Wang, Fei Bei, Haifa Hong

**Affiliations:** ^1^Department of Neonatal Intensive Care Unit, Shanghai Children's Medical Center, Shanghai Jiao Tong University School of Medicine, and National Children's Medical Center, Shanghai, China; ^2^Child Health Advocacy Institute, China Hospital Development Institute, Shanghai Jiao Tong University, Shanghai, China; ^3^Shanghai Institute for Pediatric Congenital Heart Disease, Shanghai Children's Medical Center, Shanghai Jiaotong University School of Medicine, and National Children's Medical Center, Shanghai, China; ^4^Perinatal Medicine Department of International Peace Maternal and Child Health Hospital, Shanghai Jiaotong University School of Medicine, Shanghai, China

**Keywords:** vitamin D deficiency, vasoactive inotropic score, cardiac surgery, congenital heart disease, children

## Abstract

The relationship between vitamin D and cardiovascular health in children remains unclear. Vitamin D deficiency (VDD) is supposed to be a potential risk factor associated with poorer outcomes after congenital heart disease (CHD) surgery. The maximum vasoactive-inotropic use after cardiac surgery is considered to be a good predictor of adverse outcomes. We aimed to assess the correlation between preoperative VDD and the maximum vasoactive-inotropic score (VIS_max_) at 24 h postoperatively. Nine hundred children with CHD were enrolled in this study, and preoperative total serum 25-hydroxyvitamin D [25(OH)D] concentrations were measured by liquid chromatography-tandem mass spectrometry. Related demographic and clinical characteristics were collected. A total of 490 boys (54.4%) and 410 girls (45.6%) with a mean age of 1 year (range: 6 months-3 years) were enrolled. The median 25(OH)D level was 24.0 ng/mL, with 32.6% of patients having VDD [25(OH)D < 20 ng/mL]. The univariate analysis indicated that VDD [odds ratio (OR): 2.27; 95% confidence interval (CI): 1.48–3.50] is associated with a risk of increased VISmax at 24 h postoperation. Multivariate analysis revealed that VDD (OR: 1.85; 95% CI: 1.09–3.02), a Risk-adjusted Congenital Heart Surgery score of at least three points (OR: 1.55; 95% CI: 1.09–2.19), and cardiopulmonary bypass time (OR: 1.02; 95% CI: 1.01–1.02) were independently associated with an increased VISmax within 24 h after cardiac surgery. VDD in pediatric patients before cardiac surgery is associated with the need for increased postoperative inotropic support at 24 h postoperation.

## Introduction

Congenital heart disease (CHD) is a common birth defect, found in about 1% of live births (1). It is estimated that about 130,000 new cases of CHD occur each year in China. CHD is also the leading cause of mortality among cases attributed to birth defects. Advanced cardiovascular surgery has significantly improved the survival of infants and children with CHD. The administration of vasoactive drugs postoperatively can stabilize the cardiovascular system and reduce the risk of postoperative complications. Low cardiac output syndrome (LCOS) is an important factor associated with postoperative mortality, found postoperatively in as many as 25% of infants treated for CHD (2). Treatment for LCOS consists of multiple strategies; the use of vasoactive drugs can reduce the risk of low cardiac output. The maximum vasoactive inotropic score (VIS_max_) has been used as a surrogate marker for LCOS (3). The VIS_max_ measured within 24 h after admission to the intensive care unit (ICU) is considered to be a good predictor of adverse outcomes in patients undergoing cardiac surgery (4, 5).

Mounting evidence (6–8) suggests that a close relationship exists between vitamin D and cardiovascular health. Besides regulating calcium homeostasis and maintaining skeletal health, vitamin D has a large spectrum of extraskeletal effects, such as muscle strength, regulation of the immune system, and cardiovascular health. Evidence has shown that vitamin D deficiency (VDD), characterized by a 25-hydroxyvitamin D [25(OH)D] level of <20 ng/mL, is associated with numerous adverse health outcomes in children with critical or chronic diseases. The incidence of VDD in children with CHD has been reported to be 40–84% (9). Lower vitamin D levels constitute a potential risk factor associated with prolonged mechanical ventilation (10) and longer hospital and ICU stays among critically ill children (11, 12). Lower postoperative vitamin D levels may increase the need for inotropic support (9). Meanwhile, children with preoperative VDD are at greater risk for severe VDD following cardiac surgery and cardiopulmonary bypass (CPB) (13–15). However, the association between preoperative VDD and VIS_max_ within 24 h after surgery is still unclear.

The cardiothoracic surgery division of Shanghai Children's Medical Center (SCMC, Shanghai, China) is the leading department of its kind in China and performs more than 3,000 operations per year. To date, we still know little about the vitamin D status in children with CHD in China and about the effect of VDD on postoperative clinical outcomes. The current investigation is aimed to assess the prevalence of VDD in children with CHD before surgery and its potential correlation with VIS_max_ within 24 h after surgery. We predict that VDD is common among Chinese children with CHD and that preoperative VDD increases the demand for VIS vasoactive-inotropic support within 24 h after surgery.

## Materials and Methods

### Patients and Data Collection

**Figure 1:**
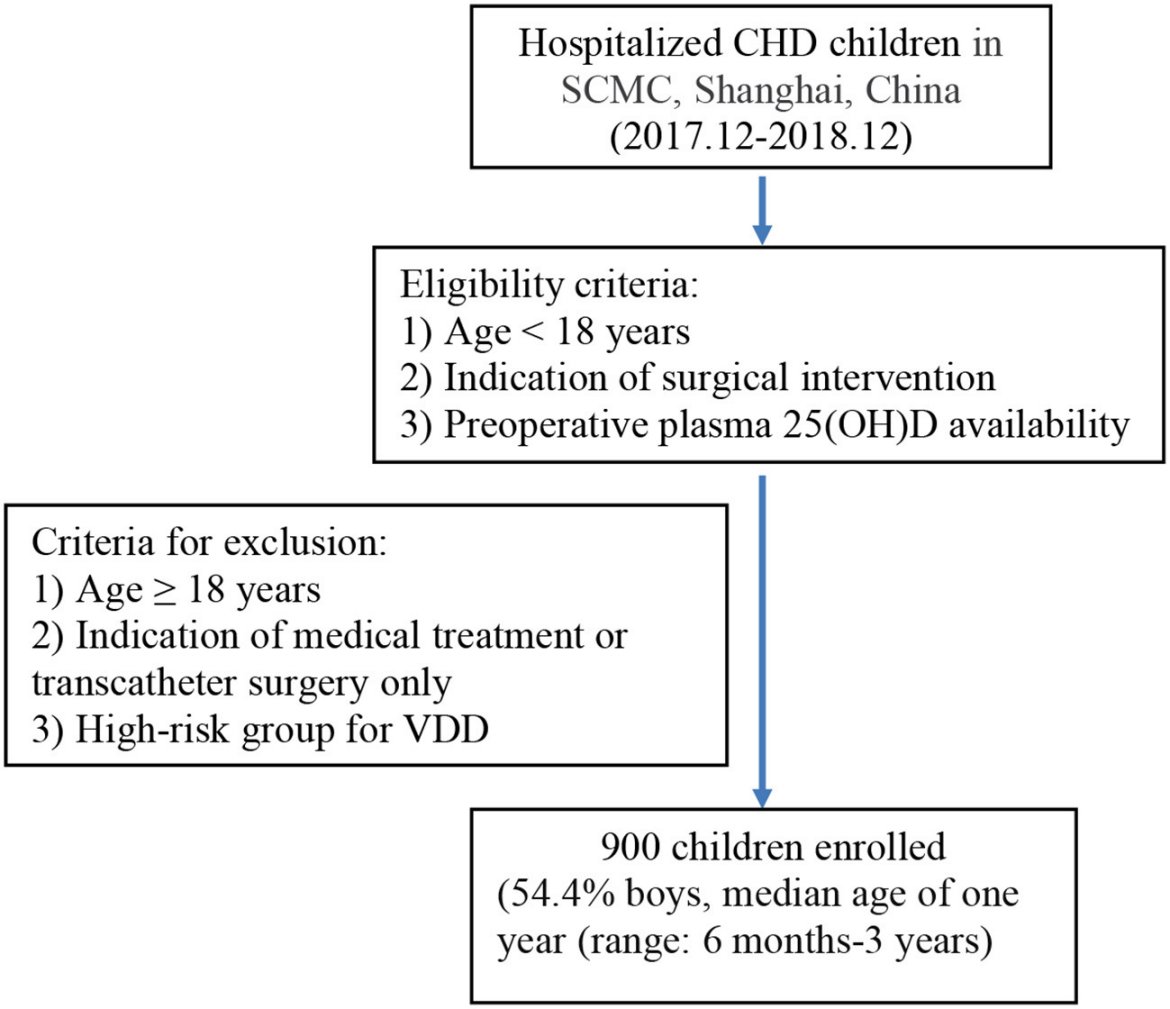
Flowchart of the study participants.

We conducted a prospective cohort study involving 900 children with CHD undergoing cardiac surgery between December 2017 and December 2018 at the SCMC, a single tertiary pediatric hospital. Eligibility criteria were the following: (1) <18 years of age, (1) indication of surgical intervention, and (3) preoperative plasma 25(OH)D availability. Criteria for exclusion were the following: (1) ≥18 years of age, (2) indication of medical treatment or transcatheter surgery only, and (3) inclusion in a high-risk group for VDD with intake of vitamin D supplementation totaling >400 IU/days within the past year, such as preterm and low-birth-weight infants (Figure 1). Liquid chromatography-tandem mass spectrometry (AB SCIEX API 3200MD; SCIEX, Concord, ON, Canada) was used to quantify the serum 25(OH)D concentration right before surgery. The study was approved by the institutional review board of the SCMC-affiliated Shanghai Jiaotong University School of Medicine (SCMCIRB-K2018039), and informed consent was obtained from the parents or guardians, as appropriate.

Relevant clinical data were collected and recorded into a computerized database. All patients were Han Chinese. We recorded demographic variables such as age, sex, and weight; details on the cardiac lesions, operative procedures, CBP duration, aortic cross-clamp time, and preoperative pneumonia were also documented. The study participants were categorized according to the Risk-adjusted Congenital Heart Surgery (RACHS) scoring system (16); there were no cases with a RACHS score of more than five points in this study. The VIS_max_ was calculated during the first 24 h post-surgery using the following equation (5): VIS = dopamine dose (μg/kg/min) + dobutamine dose (μg/kg/min) + 100 × epinephrine dose (μg/kg/min) + 10 × milrinone dose (μg/kg/min) + 10,000 × vasopressin dose (units/kg/min) + 100 × norepinephrine dose (μg/kg/min). VDD was defined as a 25(OH)D3 level of <20 ng/mL, vitamin D insufficiency was defined as a 25(OH)D3 level of 20–30 ng/mL, and vitamin D sufficiency was defined as a 25(OH)D3 level of at least 30 ng/mL (17). The cutoff for high vs. low VIS was identified as a score of 15 points during the first 24 h after heart surgery (4).

### Statistical Analysis

Statistical analysis was performed using the Statistical Package for the Social Sciences version 20 software program (IBM, Armonk, NY, USA). Continuous data were presented as median and interquartile range (IQR) values, whereas categorical data were presented as frequencies and percentages. Comparisons between groups were performed using Pearson's chi-squared (χ^2^) test for categorical data variables and the non-parametric Mann-Whitney U test for continuous variables or numerical data as appropriate. Binary logistic models were applied with VIS_max_ (<15/≥15) adopted as a response variable. Covariates were age (continuous variable), sex (female/male), vitamin D status (deficiency/insufficiency/sufficiency), season (winter/fall/spring/summer), and RACHS score class (<3/≥3) (model 2). Multivariate model 3 included the same covariates plus aortic clamping time (continuous variable) and concurrent preoperative diseases (no/yes). CPB time and clamping time were closely related to each other and could not be simultaneously put into the multivariate model. Statistical significance was considered when *p* < 0.05.

## Results

A total of 900 Chinese children [(490 boys (54.4%) and 410 girls (45.6%)] with a median age of 1 year (range: 6 months-3 years) were included in this study. The median 25(OH)D level of the enrolled children was 24.0 ng/mL (IQR: 17.9–29.4); the plasma 25(OH)D level was below 20 ng/mL in 32.6% of children (*n* = 293), from 20 to 30 ng/mL in 44.4% of children (*n* = 400), and above 30 ng/mL in 23% of children (*n* = 207). No significant differences were detected in the demographic data of the patients, including sex, body mass index (BMI), season, and preoperative pneumonia, among the three 25(OH)D groups (*p* > 0.05). However, there were significant differences in age (*p* < 0.001), CPB time (*p* < 0.001), clamping time (*p* < 0.001), RACHS score of at least three points (*p* = 0.004), and VIS_max_ (≥15 points) within the first 24 h after surgery (*p* < 0.001) (Table 1).

**Table 1:** Demographic and clinical characteristics of the study participants according to preoperative vitamin D status.

**Parameter**	**Total**	**25(OH)D <20 ng/mL (*n =* 293)**	**25(OH)D = 20-30 ng/mL (*n =* 400)**	**25(OH)D ≥ 30 ng/mL (*n =* 207)**	**Z/χ^2^**	***p*-value**
Age, years, median (IQR)	1.0 (0.5–3.0)	2.4 (0.4–5.3)	1.0 (0.4–2.3)	0.7 (0.5–1.2)	56.93	0.000
Male sex, *n* (%)	490 (54.4)	159 (54.3)	212 (53.0)	119 (57.5)	1.11	0.573
Season	900 (100.0)	293 (32.6)	400 (44.4)	207 (23.0)	8.10	0.231
Spring, *n* (%)	282 (31.3)	100 (34.1)	117 (29.3)	65 (31.4)		
Summer, *n* (%)	283 (31.4)	91 (31.1)	126 (31.5)	66 (31.9)		
Fall, *n* (%)	167 (18.6)	41 (14.0)	82 (20.5)	32 (21.3)		
Winter, *n* (%)	168 (18.7)	61 (20.8)	75 (18.8)	32 (15.5)		
BMI, median (IQR)	15.6 (14.4–16.8)	15.6 (14.2–16.8)	15.6 (14.4–16.8)	15.7 (14.6–16.9)	1.12	0.570
CPB time (min), median (IQR)	56.0 (40.0–85.7)	67.0 (45.0–67.0)	52.0 (40.0–79.0)	50.0 (37.0–75.0)	30.19	0.000
Clamping time (min), median (IQR)	30.0 (20.0–51.0)	37.0 (21.5–68.5)	29.0 (19.0–48.0)	26.0 (19.0–43.0)	16.82	0.000
RACHS score, *n* (%)					10.89	0.004
≥3	305 (33.9)	121 (41.3)	124 (31.0)	60 (29.0)		
<3	595 (66.1)	172 (58.7)	276 (69.0)	147 (71.0)		
Preoperative pneumonia, *n* (%)	27 (3.0)	12 (4.1)	12 (3.0)	3 (1.4)	2.92	0.232
VIS, *n* (%)					16.95	0.000
≥15	225 (25.0)	97 (33.1)	91 (22.8)	37 (17.9)		
<15	675 (75.0)	196 (66.9)	309 (77.3)	170 (82.1)		

*IQR, interquartile range; CPB, cardiopulmonary bypass time; RACHS, Risk-adjusted Congenital Heart Surgery; VIS, vasoactive-inotropic score*.

There was no significant difference in age, sex, BMI, seasonal distribution, or preoperative pneumonia between the two VIS score groups according to the 15-point cutoff (*p* > 0.05). There were, however, significant differences in CPB time (*p* < 0.001), clamping time (*p* < 0.001), and RACHS score of at least three points (*p* < 0.001) (Table 2).

**Table 2:** Demographic and clinical characteristics of study participants according to postoperative VIS_max_ during the first 24 h after surgery.

**Parameter**	**Total (*n =* 900)**	**VIS <15 points (*n =* 675)**	**VIS ≥ 15 points (*n =* 225)**	**Z/χ^**2**^**	***p*-value**
Age, years, median (IQR)	1.0 (0.5–3.0)	1.1 (0.5–2.8)	0.8 (0.4–3.9)	−0.72	0.469
Male sex, *n* (%)	490 (54.4)	356 (52.7)	134 (59.6)	3.16	0.075
Season				5.64	0.130
Spring, *n* (%)	282 (31.3)	215 (31.9)	67 (29.8)		
Summer, *n* (%)	283 (31.4)	223 (33.0)	60 (26.7)		
Fall, *n* (%)	167 (18.6)	118 (17.5)	49 (21.8)		
Winter, *n* (%)	168 (18.7)	119 (17.6)	49 (21.8)		
BMI, median (IQR)	15.6 (14.4–16.8)	15.6 (14.5–16.8)	15.6 (14.3–16.9)	−1.11	0.268
CPB time, min, median (IQR)	56.0 (40.0–85.7)	50.0 (38.0–70.0)	93.0 (65.0–133.5)	−11.99	0.000
Clamping time, min, median (IQR)	30.0 (20.0–51.0)	26.0 (18.0–39.0)	55.0 (31.5–78.5)	−10.71	0.000
RACHS score, *n* (%)				31.94	0.000
≥3	305 (33.9)	194 (28.7)	111 (49.3)		
<3	595 (66.1)	481 (71.3)	114 (50.7)		
Preoperative pneumonia, *n* (%)	27 (3.0)	17 (2.5)	10 (4.4)	2.15	0.142

*IQR, interquartile range; CPB, cardiopulmonary bypass time; VIS_max_, maximum vasoactive inotropic score; RACHS, Risk-adjusted Congenital Heart Surgery*.

The univariate analysis indicated that the following factors were associated with an increased risk of VIS_max_ at 24 h postoperation: VDD [odds ratio (OR): 2.27; 95% confidence interval (CI): 1.48–3.50], RACHS score of at least three points (OR: 2.4; 95% CI: 1.77–3.29), CPB time (OR: 1.02; 95% CI: 1.02–1.03), and clamping time (OR: 1.02; 95% CI: 1.02–1.03). Model 2 showed that VDD and a RACHS score of at least three points were independently associated with an increased VIS_max_ at 24 h postoperation (OR: 2.14; 95% CI: 1.33–3.43 and OR: 2.31; 95% CI: 1.68–3.19, respectively). Model 3 showed that VDD (OR: 1.85; 95% CI: 1.10–3.02), a RACHS score of at least three points (OR: 1.55; 95% CI: 1.09–2.19), and CPB time (OR: 1.02; 95% CI: 1.01–1.02) were independently linked to an increased VIS_max_ at 24 h postoperation (Table 3).

**Table 3:** Logistic regression analysis of the risk factors for VIS_max_ within 24 h after surgery.

	**Model 1^a^**	**Model 2^b^**	**Model 3^c^**
Age	1.051 (0.998–1.107)	1.002 (0.945–1.063)	0.955 (0.892–1.021)
Female sex	0.758 (0.558–1.029)	0.746 (0.544–1.024)	0.747(0.531–1.051)
Vitamin D level			
25(OH)D = 20–30 ng/mL	1.353 (0.884–2.071)	1.355 (0.876–2.096)	1.336 (0.843–2.120)
25(OH)D <20 ng/mL	2.274 (1.478–3.498)	2.141 (1.335–3.434)	1.819 (1.096–3.020)
Season			
Spring	0.757 (0.492–1.165)	0.757 (0.485–1.182)	0.731 (0.451–1.183)
Summer	0.653 (0.422–1.013)	0.646 (0.411–1.016)	0.686 (0.420–1.123)
Fall	1.008 (0.630–1.615)	1.052 (0.640–1.729)	1.159 (0.675–1.991)
RACHS score ≥3 points	2.414 (1.771–3.291)	2.309 (1.683–3.168)	1.546 (1.090–2.193)
CPB time	1.020 (1.016–1.024)	-	1.019 (1.015–1.023)
Clamping time	1.023 (1.018–1.028)	-	-
Preoperative pneumonia	1.800 (0.812–3.991)	-	0.956 (0.392–2.336)

*Values are presented as OR (95% CI)*.

*CPB, cardiopulmonary bypass time; RACHS, the Risk-adjusted Congenital Heart Surgery; VIS_max_, maximum vasoactive inotropic score*.

a *Univariate logistic regression model*.

b *Multivariate logistic regression model in which age, sex, vitamin D class, season, and RACHS were controlled*.

c *Multivariate logistic regression model in which age, sex, vitamin D status, season, RACHS score, CPB time, and concurrent preoperative diseases were controlled. CPB time and clamping time were closely related to each other and could not be put into the model at the same time*.

## Discussion

A high prevalence of VDD in children has been observed worldwide. Studies have reported that the prevalence of VDD ranges from 40 to 49.3% in children with CHD (9, 18). In our study, VDD was observed in 32.6% of the children, a rate that is a little lower than that mentioned in prior literature reports. A possible reason for this result is the relatively young age of our study population at baseline, with 75% of the children aged younger than 3 years old. China's health care system guidance has identified the need for vitamin D supplementation in certain at-risk groups, particularly infants (19). We also found that children with VDD most often experience longer CPB and clamping times, while the proportion of VDD is higher in children with RACHS scores of at least three points. However, no significant difference in vitamin D level according to sex, season, BMI, or preoperative pneumonia was found. Studies showed that CPB is associated with a reduction in vitamin D levels in pediatric patients following cardiac surgery (9, 18). As a part of this study, serial vitamin D measurements were performed in 21 children with tetralogy of Fallot, and we found that CPB significantly affected the level of 25(OH)D after surgery in this subgroup (20). The decrease in serum 25(OH)D level is most likely explained by hemodilution due to acute fluid shift, and CPB tubing or oxygenator membrane absorption, blood loss, or inflammation also represent other potential reasons for the decline (9). The incidence of VDD after cardiac surgery may be higher in children with longer CPB and clamping times. The RACHS score relies on subjective assessments of operative risk and cardiac anatomy by congenital heart surgeons and pediatric cardiologists, with higher RACHS scores associated with a greater in-hospital mortality risk (16). The children with higher RACHS scores not only have primary VDD but also suffer from secondary VDD, requiring longer CPB and clamping times (21).

A growing number of studies have suggested that VDD influences the outcome of CHD surgery (22, 23). In our study, 12 patients died before discharge, resulting in a postoperative mortality rate of 1.3%. LCOS is an important factor related to postoperative mortality. The use of vasoactive drugs can reduce the risk of LCOS. Gaies et al. suggested that the VIS_max_ within 24 h after admission to the cardiac ICU can be used as a predictor of adverse prognosis after cardiac surgery (4, 5). In our study, 25% of children required greater VIS (≥15 points) support. Children with VDD or prolonged CPB and clamping times during surgery typically require greater VIS support. It seems that CPB and aortic clamping times were longer in children with higher VIS_max_ values and such may be the cause of the need for greater vasoactive-inotropic drug support independently of VDD. However, the association remained significant after adjusting for these variables, which point to an independent association between VDD and high VIS_max_ (OR: 2.14; 95% CI: 1.33–3.43). Our analysis demonstrates that preoperative VDD was associated with a greater need for inotropic support in CHD children.

Low vitamin D levels could directly influence cardiac myocytes and endothelial function through cellular vitamin D receptors (8). Our study's results strongly support the need for deploying more vasoactive drugs in children with CHD and VDD before surgery. A more severe VDD status is typically found after surgery and CPB in children who already had VDD before surgery (13–15). Lower postoperative 25(OH)D levels may increase the need for vasoactive drug support (9). In our study, children with low VIS values had a mean CPB time of 50 min and children with high VIS values had a mean CPB time of 93 min. This may contribute to increased use of vasoactive-inotropic support postoperatively. Meanwhile, maintaining adequate postoperative vitamin D levels following surgery may speed up patient recovery following CHD surgery (13, 24). Thus, the requirements for vitamin D supplementation in patients with CHD may differ from those in healthy children; however, to our knowledge, the benefits of vitamin D supplementation for CHD surgery have not been confirmed. Recommendations regarding a proper vitamin D dose in CHD children have not been offered and no consistent conclusions have been reached to date (25, 26).

Our study has many strengths and some limitations. The potential strengths are our large sample size, analysis of plasma 25(OH)D using high-quality methods, the fact that the findings arose from multivariate analyses adjusted using key confounders for vitamin D status. However, there are still some limitations to our research. First, the main limitation is the inclusion of children of one ethnicity, which limits the comparability with data gleaned from other ethnicities, primarily ethnic minority groups living far away from Shanghai. Distance is an important factor in hospital choice in that people are more likely to choose the hospitals that are located close to their homes. Second, we did not record serial vitamin D measurements following CPB, although the literature has also indicated that lower postoperative vitamin D levels may be associated with the need for greater inotropic support (9). Serial vitamin D measurements of five time points were collected from a small sample of 21 children with tetralogy of Fallot (20). We found that CPB significantly affected the level of 25(OH)D after surgery. Unfortunately, there were no significant differences between lower postoperative vitamin D levels (both right after surgery and 24 h postoperation) and VIS_max_ within 24 h after surgery. Third, this study only recorded the VIS_max_ within 24 h after surgery, while that at 48 h was not recorded. Some studies (3, 27) have shown that the VIS_max_ at 48 h may be more representative of the patient's prognosis. Additional research is needed to better understand the relationship between preoperative 25(OH)D level and VIS_max_ at 48 h after surgery. Fourth and finally, the study did not examine the correlation between preoperative VDD and short- and long-term clinical outcomes after heart surgery.

## Conclusions

This study provides clear evidence that VDD is prevalent among Chinese children with CHD following cardiac surgery in China. Importantly, lower vitamin D levels before pediatric cardiac surgery are associated with a greater need for inotropic support. Further research is needed.

## Data Availability Statement

The original contributions presented in the study are included in the article/supplementary material, further inquiries can be directed to the corresponding author/s.

## Ethics Statement

The studies involving human participants were reviewed and approved by the institutional review board of Shanghai Children's Medical Center affiliated Shanghai Jiaotong University School of Medicine (SCMCIRB-K2018039). Written informed consent to participate in this study was provided by the participants' legal guardian/next of kin.

## Author Contributions

XY, YW, FB, and HH made substantial contributions to the conception and design of the study. XY, SD, YD, CJ, YK, and LT contributed to the acquisition, analysis, or interpretation of the data. XY and SD performed the statistical analysis. XY drafted the article and helped perform the analysis with constructive discussions. All the authors have read and approved the final version to be published.

## Conflict of Interest

The authors declare that the research was conducted in the absence of any commercial or financial relationships that could be construed as a potential conflict of interest.

## Publisher's Note

All claims expressed in this article are solely those of the authors and do not necessarily represent those of their affiliated organizations, or those of the publisher, the editors and the reviewers. Any product that may be evaluated in this article, or claim that may be made by its manufacturer, is not guaranteed or endorsed by the publisher.

## References

[1] Triedman JK Newburger JW. Trends in congenital heart disease: the next decade. Circulation. (2016) 133:2716–33. 10.1161/CIRCULATIONAHA.116.023544 27324366

[2] Stocker CF Shekerdemian LS. Recent developments in the perioperative management of the paediatric cardiac patient. Curr Opin Anaesthesiol. (2006) 19:375–81. 10.1097/01.aco.0000236135.77733.cd 16829717

[3] Davidson J Tong S Hancock H Hauck A da Cruz E Kaufman J. Prospective validation of the vasoactive-inotropic score and correlation to short-term outcomes in neonates and infants after cardiothoracic surgery. Intensive Care Med. (2012) 38:1184–90. 10.1007/s00134-012-2544-x 22527067 PMC4984395

[4] Gaies MG Jeffries HE Niebler RA Pasquali SK Donohue JE Yu S. Vasoactive-inotropic score is associated with outcome after infant cardiac surgery: an analysis from the pediatric cardiac critical care consortium and virtual pICU system registries. Pediatr Crit Care Med. (2014) 15:529–37. 10.1097/PCC.0000000000000153 24777300 PMC4159673

[5] Gaies MG Gurney JG Yen AH Napoli ML Gajarski RJ Ohye RG. Vasoactive-inotropic score as a predictor of morbidity and mortality in infants after cardiopulmonary bypass. Pediatr Crit Care Med. (2010) 11:234–8. 10.1097/PCC.0b013e3181b806fc 19794327

[6] Alonso MA Mantecón L Santos F. Vitamin D deficiency in children: a challenging diagnosis! Pediatr Res. (2019) 85:596–601. 10.1038/s41390-019-0289-8 30653195

[7] Ohlund I Lind T Hernell O Silfverdal SA Liv P Karlsland Akeson P. Vitamin d status and cardiometabolic risk markers in young Swedish children: a double-blind randomized clinical trial comparing different doses of vitamin d supplements. Am J Clin Nutr. (2020) 111:779–86. 10.1093/ajcn/nqaa031 32140704 PMC7138658

[8] Pilz S Verheyen N Grubler MR Tomaschitz A Marz W. Vitamin d and cardiovascular disease prevention. Nat Rev Cardiol. (2016) 13:404–17. 10.1038/nrcardio.2016.73 27150190

[9] Dohain AM Almogati J Al-Radi OO Elassal AA Zaher ZF Fatani TH. Serum vitamin d status following pediatric cardiac surgery and association with clinical outcome. Eur J Pediatr. (2020) 179:635–43. 10.1007/s00431-019-03538-x 31865429

[10] Razavi Khorasani NMB Zahedi Tajrishi F Mohammadpour Z Rouhi F Alizadeh-Navaei R Ghadimi R. The association between low levels of vitamin d and clinical outcomes in critically-Ill children: a Systematic review and meta-Analysis. Fetal Pediatr Pathol. (2020) 39:503–17. 10.1080/15513815.2019.1675832 31603014

[11] McNally JD Nama N O'Hearn K Sampson M Amrein K Iliriani K. Vitamin d deficiency in critically ill children: a systematic review and meta-analysis. Crit Care. (2017) 21:287. 10.1186/s13054-017-1875-y 29169388 PMC5701429

[12] Dao DT Anez-Bustillos L Cho BS Li Z Puder M Gura KM. Assessment of micronutrient status in critically ill children: challenges and opportunities. Nutrients. (2017) 9:1185. 10.3390/nu9111185 29143766 PMC5707657

[13] McNally JD O'Hearn K Lawson ML Maharajh G Geier P Weiler H. Prevention of vitamin d deficiency in children following cardiac surgery: study protocol for a randomized controlled trial. Trials. (2015) 16:402. 10.1186/s13063-015-0922-8 26353829 PMC4564959

[14] Krishnan A Ochola J Mundy J Jones M Kruger P Duncan E. Acute fluid shifts influence the assessment of serum vitamin D status in critically ill patients. Crit Care. (2010) 14:R216. 10.1186/cc9341 21110839 PMC3219984

[15] McNally JD Menon K Chakraborty P Fisher L Williams KA Al-Dirbashi OY. Impact of anesthesia and surgery for congenital heart disease on the vitamin d status of infants and children: a prospective longitudinal study. Anesthesiology. (2013) 119:71–80. 10.1097/ALN.0b013e31828ce817 23470437

[16] Jenkins KJ Gauvreau K Newburger JW Spray TL Moller JH Iezzoni LI. Consensus-based method for risk adjustment for surgery for congenital heart disease. J Thorac Cardiovasc Surg. (2002) 123:110–8. 10.1067/mtc.2002.119064 11782764

[17] Pearce SH Cheetham TD. Diagnosis and management of vitamin d deficiency. BMJ. (2010) 340:b5664. 10.1136/bmj.b5664 20064851

[18] Abou Zahr R Faustino EVS Carpenter T Kirshbom P Hall EK Fahey JT. Vitamin d Status after cardiopulmonary bypass in children with congenital heart disease. J Intensive Care Med. (2017) 32:508–13. 10.1177/0885066616652077 27251108

[19] Bouillon R. Comparative analysis of nutritional guidelines for vitamin d. Nat Rev Endocrinol. (2017) 13:466–79. 10.1038/nrendo.2017.31 28387318

[20] Ye X Jiang C Hong H Sun J Bu J Hong H. Peri-operative vitamin d status and its association with postoperative clinical outcome in children with tetralogy of fallot. [in Chinese] *J Bio-education*. (2020) 8:106–9. 10.3969/j.issn.2095-4301.2020.02.004

[21] McNally JD Menon K. Vitamin d deficiency in surgical congenital heart disease: prevalence and relevance. Transl Pediatr. (2013) 2:99–111. 10.3978/j.issn.2224-4336.2013.07.03 26835300 PMC4728932

[22] Ney J Heyland DK Amrein K Marx G Grottke O Choudrakis M. The relevance of 25-hydroxyvitamin d and 1,25-dihydroxyvitamin D concentration for postoperative infections and postoperative organ dysfunctions in cardiac surgery patients: the eVIDenCe study. Clin Nutr. (2019) 38:2756–62. 10.1016/j.clnu.2018.11.033 30583965

[23] Zittermann A KJ Ernst JB Becker T Dreier J Knabbe C Gummert JF. 25-hydroxyvitamin D, 1,25-dihydroxyvitamin D and postoperative outcome in cardiac surgery. J Clin Endocrinol Metab. (2015) 100:72–80. 10.1210/jc.2014-3013 25365313

[24] McNally JD O'Hearn K Fergusson DA Lougheed J Doherty DR Maharajh G. Prevention of post-cardiac surgery vitamin D deficiency in children with congenital heart disease: a pilot feasibility dose evaluation randomized controlled trial. Pilot Feasibility Stud. (2020) 6:159. 10.1186/s40814-020-00700-3 33110622 PMC7583219

[25] National Heart L Blood Institute PCTN Ginde AA Brower RG Caterino JM Finck L. Early high-dose vitamin d3 for critically ill, vitamin d-Deficient patients. N Engl J Med. (2019) 381:2529–40. 10.1056/NEJMoa1911124 31826336 PMC7306117

[26] Zittermann A Ernst JB Prokop S Fuchs U Dreier J Kuhn J. Effect of vitamin d on all-cause mortality in heart failure (EVITA): a 3-year randomized clinical trial with 4000 iU vitamin d daily. Eur Heart J. (2017) 38:2279–86. 10.1093/eurheartj/ehx235 28498942

[27] Dilli D Akduman H Orun UA Tasar M Tasoglu I Aydogan S. Predictive value of vasoactive-inotropic score for mortality in newborns undergoing cardiac surgery. Indian Pediatr. (2019) 56:735–40. 10.1007/s13312-019-1639-7 31638004

